# Optimization of a Method for the Simultaneous Extraction of Polar and Non-Polar Oxylipin Metabolites, DNA, RNA, Small RNA, and Protein from a Single Small Tissue Sample

**DOI:** 10.3390/mps3030061

**Published:** 2020-08-26

**Authors:** Yu Hasegawa, Yurika Otoki, Shannon McClorry, Laurynne C. Coates, Rachel L. Lombardi, Ameer Y. Taha, Carolyn M. Slupsky

**Affiliations:** 1 Department of Food Science and Technology, University of California-Davis, Davis, CA 95616, USA; yhasegawa@ucdavis.edu (Y.H.); yurika.otoki.d7@tohoku.ac.jp (Y.O.); lnchetelat@ucdavis.edu (L.C.C.); rllombardi@ucdavis.edu (R.L.L.); ataha@ucdavis.edu (A.Y.T.); 2 Graduate School of Agricultural Science, Tohoku University, Sendai, Miyagi 980-8572, Japan; 3 Department of Nutrition, University of California-Davis, Davis, CA 95616, USA; svmcclorry@ucdavis.edu

**Keywords:** molecular biology, analytical chemistry, DNA, RNA, protein, metabolites, extraction, purification, tissue, systems biology

## Abstract

A more comprehensive picture of tissue biology can be obtained through the application and integration of multiple omic technologies. However, the common challenge in working with a precious sample is having a sample too small to separately extract analytes of interest for each experiment. Considering the high heterogeneity that can be present in a single tissue sample, extracting all biomolecules from a single and undivided tissue is preferable because it allows direct comparison of results. Here, we combined a modified Folch extraction method with DNA, RNA, small RNA, and protein extraction using two commercial kits, which allowed us to extract polar metabolites and non-polar oxylipin metabolites, DNA, RNA, small RNA, and protein simultaneously from a small tissue sample. The method was validated in terms of quantity and quality of analytes for downstream analyses.

## 1. Introduction

Multi-omic studies allow us to capture a comprehensive picture of the biology of a system. However, when tissue is limited in size, such as a specific brain region in a mouse, one must choose the analyte of interest. Even when there is enough sample to perform all analyses of interest, measuring all biomolecules derived from a single sample is preferable to avoid potential variation that could be introduced by studying specimens divided and prepared separately [1], as there is often heterogeneity in different regions of a tissue [2].

**Figure 1:**
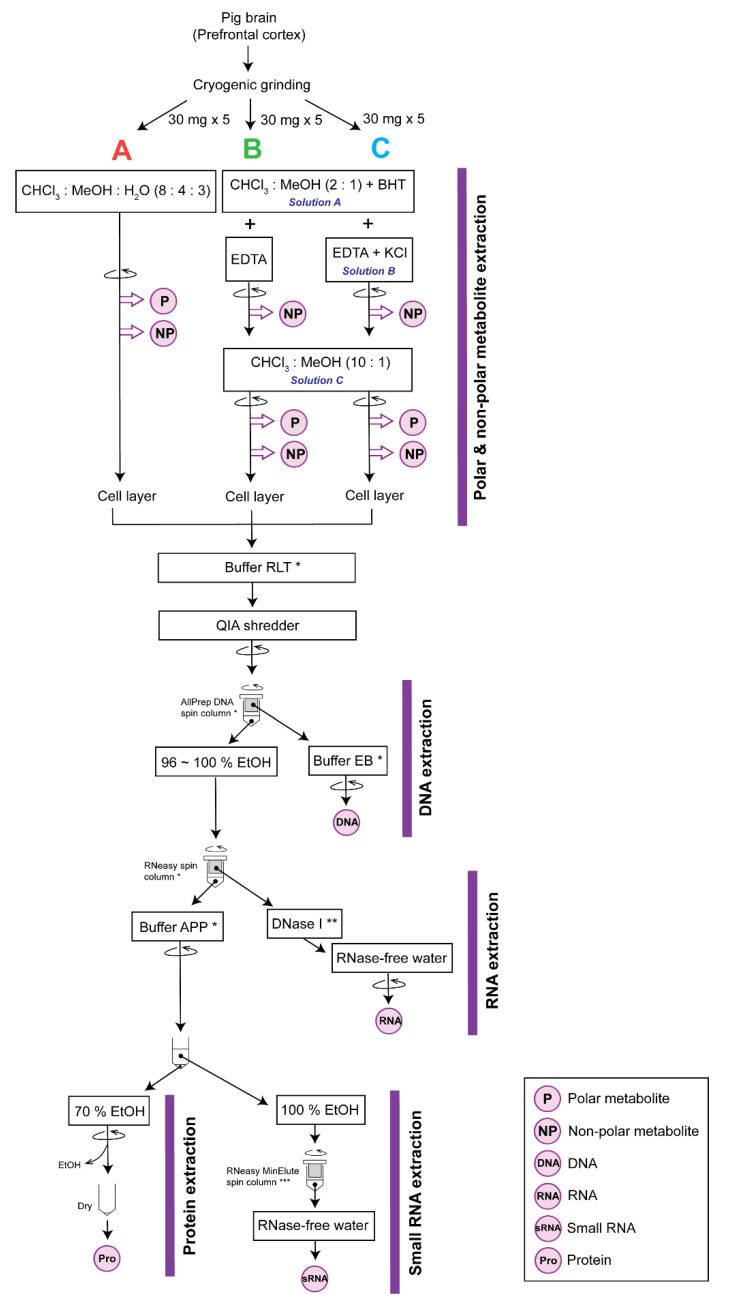
Overall schematic of the methods. Method A is the method proposed by Roume et al. [1]. Reagents with * are from the AllPrep DNA/RNA/Protein mini kit, ** from the RNase-Free DNase Set, and *** from the RNeasy MinElute cleanup kit. The round arrow indicates that centrifugation was applied at that step. Extracted analytes are indicated with purple circles. Abbreviations: CHCl3, chloroform; MeOH, methanol; H2O, water; EtOH, ethanol.

Previously, Roume et al. [1] reported a method that allows the extraction of polar and non-polar metabolites, DNA, RNA, small RNA, and protein from a single sample. However, we found this method to have three major limitations. First, the reported method was not optimized for non-polar metabolites of polyunsaturated fatty acids that have low stability and concentration, such as oxylipins. Second, we found that some proteins required further purification to be detected by Western blot. Third, we found high variability in the reported method. Here, we report on a modified multi-extraction method that improves the isolation and reproducibility of extraction of biomolecules from a tissue sample as small as 30 mg. We ground pre-frontal cortex of a pig brain under liquid nitrogen to a fine, homogeneous powder and aliquoted it such that five technical replicates for each of three methods could be tested (Figure 1). Method A corresponded to the method proposed by Roume et al. [1]. Methods B and C differed from Method A in terms of the addition of ethylenediaminetetraacetic acid (EDTA) and butylated hydroxytoluene (BHT) in water, and an additional extraction step. Method C differed from Method B in that the extraction mixture for Method C also contained potassium chloride (KCl). Considering the reproducibility, quantity, and quality of the extracts, we concluded that Method C produced the best results, which is proposed in this paper. The protocols for Methods A and B are summarized in the Supplementary Information.

## 2. Experimental Design

### 2.1. Materials

#### 2.1.1. Tissue Preparation

Six-week-old piglets were anesthetized with an intramuscular injection of 0.04 mL/kg of a solution containing Telazol (100 mg/mL), ketamine (50 mg/mL), and xylazine (50 mg/mL), then euthanized with an overdose of pentobarbital via intracardiac injection (0.22 mL/kg) (FatalPlus, Vortech Pharmaceuticals, Dearborn, MI, USA). All animal procedures were approved by the Institutional Animal Care and Use Committee at the University of California-Davis (protocol #20148). Following euthanasia, the pig prefrontal cortex was collected and snap-frozen in liquid nitrogen. The brain tissue was stored at −80 °C until further analyses to avoid any enzymatic reactions or disruption of analytes.

#### 2.1.2. Extraction

- Chloroform (HPLC-grade; Fisher Scientific, Waltham, MA, USA)
- Methanol (HPLC-grade; Fisher Scientific)
- Type I ultrapure water
- BHT (Sigma–Aldrich, St. Louis, MO, USA)
- EDTA (Fisher Scientific)
- KCl (Crystalline/Certified ACS; Fisher Scientific)

#### 2.1.3. Polar Metabolite Analysis

- 10 mM phosphate buffer (pH 6.85)
- 3-(trimethylsilyl)-1-propanesulfonic acid-d6 (DSS-d6) containing 0.2% NaN3 in 98% deuterium oxide (Chenomx Inc., Edmonton, AB, Canada)
- Hydrochloric acid (HCl)
- Sodium hydroxide (NaOH)

#### 2.1.4. Non-polar Oxylipin Metabolite Analysis

- Isopropanol (Fisher Scientific)
- Surrogate standard solution
- Triphenylphosphine
- Sodium carbonate
- Acetic acid
- Ethyl acetate
- Acetonitrile

#### 2.1.5. Cell Lysis and Extraction of DNA, RNA, Small RNA, and Protein

- 2-mercaptoethanol (Sigma–Aldrich)
- AllPrep DNA/RNA/Protein Mini Kit (QIAGEN, Germantown, MD, USA)
- QIAshredder (QIAGEN)
- RNase-Free DNase Set (QIAGEN)
- RNeasy MinElute Cleanup Kit (QIAGEN)
- Ethanol (Decon Labs)
- 5% w/v sodium dodecyl sulfate (SDS) (Sigma–Aldrich)
- Bovine serum albumin (Bio-Rad, Hercules, CA, USA)

### 2.2. Equipment

#### 2.2.1. Extraction

- Balance
- Biosafety cabinet
- Fume hood
- Pipettes
- DNA AWAY surface decontaminant (Thermo Fisher Scientific, Waltham, MA, USA)
- RNaseZap RNase decontamination solution (Thermo Fisher Scientific)
- Liquid nitrogen
- Sterile razors
- Aluminum foil
- Dry ice
- Ice
- Mortar and pestles
- Disposable standard spoon/spatula (USA Scientific, Ocala, FL, USA)
- 8 mL glass tubes (Fisher Scientific)
- Falcon 15 mL conical centrifuge tubes (Fisher Scientific)
- 9-inch glass Pasteur pipettes (Fisher Scientific)
- Vortexer
- Benchtop centrifuge for 1.5 mL microcentrifuge tubes
- 5 mL tubes
- Benchtop centrifuge for 8 mL glass tubes/15 mL falcon tubes
- Dry baths/block heater (Fisher Scientific)

#### 2.2.2. Polar Metabolite Analysis

- 1.5 mL microcentrifuge tubes
- MiVac Duo concentrator (Genevac Ltd., Ipswich, England)
−80 and −20 °C freezers
- 4 °C refrigerator
- pH meter
- 3 mm nuclear magnetic resonance (NMR) tube (Bruker, Billerica, MA, USA)
- Bruker Avance 600 MHz NMR spectrometer (Bruker)

#### 2.2.3. Non-Polar Oxylipin Metabolite Analysis

- Waters Oasis HLB 3cc SPE column (Waters, Milford, MA, USA)
- Agilent Eclipse Plus C18 column (2.1 × 150 mm, 1.8 μm) (Agilent Corporation, Folsom, CA, USA)
- Agilent LC system 1290 coupled to an Agilent 6460 Triple Quadrupole mass-spectrometer (Agilent Corporation)

#### 2.2.4. DNA, RNA, Small RNA Assessment

- NanoDrop 2000c spectrophotometer (Thermo Fisher Scientific)
- Qubit Fluorometer 3.0 (Thermo Fisher Scientific)
- Qubit dsDNA BR Assay Kit (Thermo Fisher Scientific)
- Qubit RNA HS Assay Kit (Thermo Fisher Scientific)
- Qubit microRNA Assay Kit (Thermo Fisher Scientific)
- Gel loading dye purple (6X) containing no SDS (New England BioLabs, Ipswich, MA, USA)
- SYBR Safe DNA Gel Stain (Thermo Fisher Scientific)
- Gel Doc XR system (Bio-Rad)
- Agilent 2100 Bioanalyzer (Agilent)
- Agilent RNA 6000 Nano Kit (Agilent)

#### 2.2.5. Protein Assessment

- DC Protein Assay kit (Bio-Rad)
- Synergy H1 hybrid multi-mode reader (BioTek Instruments, Winooski, VT, USA)
- 96-well plate

#### 2.2.6. Further Protein Purification

- Zeba Spin desalting columns, 7K MWCO (Thermo Fisher Scientific)

## 3. Procedure

### 3.1. Preparation of Reagents. Time for Completion: 0:10 Hours per Batch of 12 Samples

1. Prepare chloroform:methanol (2:1) with 0.002% BHT [Solution A]. Pre-chill in a −20 °C freezer.
2. Prepare 1 mM EDTA dissolved in Type I water with 0.9% KCl [Solution B]. Pre-chill to 4 °C.
3. Prepare chloroform:methanol (10:1) [Solution C]. Pre-chill in a −20 °C freezer.

### 3.2. Tissue Disruption. Time for Completion: 2:00 Hours per Batch of 12 Samples

In order to disrupt the tissue, a mortar and pestle was used in this protocol, which may be replaced by a rotor–stator homogenizer or bead-milling. However, the long and intense homogenization time incurred by using these methods can lead to fragmentation of genomic DNA and the degradation of protein due to the heat generated. In addition, some commercial beads may not be used as they react with lysis buffer used in this protocol (Buffer RLT). Cryogrinding can have sample loss of 10–20 mg, depending on the researcher’s skill. Although as small as 10 mg of cryoground tissue can achieve good quality extracts, it is recommended to start with a tissue size of 40–50 mg in order to secure ~30 mg.

4. Prior to tissue homogenization and extractions, clean all equipment by either autoclaving or spraying with DNA AWAY surface decontaminant and RNaseZap RNase decontamination solution. All disposable supplies should be purchased sterilized and free of DNase and RNase.
5. Weigh glass tubes with lids.
6. In a biosafety cabinet (BSC), cut tissue using a sterile razor on aluminum foil that has been placed on dry ice.
7. In the BSC, place the tissue into a mortar pre-chilled with liquid nitrogen and grind with a pre-chilled pestle until a frozen, homogeneous, and fine powder is obtained, adding additional liquid nitrogen as needed.
8. In the BSC, chill a disposable spatula with liquid nitrogen, and transfer 30 mg of the cryoground tissue into a pre-weighed 8 mL glass tube.

### 3.3. Metabolite Extraction. Time for Completion: 1:20 Hours per Batch of 12 Samples

9. Add 2.4 mL of cold Solution A into the 8 mL glass tube with the cryoground tissue.
10. Add 600 μL of Solution B.
11. Cap carefully and vortex for 20 s at max speed, and centrifuge for 15 min at 2000× *g* at 0 °C.
12. Using a 9-inch glass Pasteur pipette, collect the bottom layer and place in a new 8 mL glass tube. Place the tube on ice.
13. Add 1.4 mL of cold Solution C to the remaining upper and middle layer.
14. Vortex for 10 s at max speed, and centrifuge for 15 min at 2000× *g* at 0 °C.
15. Collect the upper layer into a new 15 mL conical centrifuge tube. Do not disturb the middle layer. Keep the tube on ice.
16. Collect the bottom layer with a Pasteur pipette, and add to the glass tube from step 12. Keep both tubes on ice.

### 3.4. Cell Lysis and Homogenization. Time for Completion: 0:20 Hours per Batch of 12 Samples

17. In a fume hood, modify the Buffer RLT provided from a QIAGEN AllPrep DNA/RNA/Protein Mini Kit by adding 10 µL of 2-mercaptoethanol for each mL of Buffer RLT. Wrap the cap with parafilm and pre-chill the buffer at 4 °C for at least 10 min before use.

- Buffer RLT contains guanidine thiocyanate. The combination of guanidine thiocyanate with 2-mercaptoethanol will ensure the inactivation of enzymes such as RNase as well as proteinases. The modified Buffer RLT can be stored at room temperature for up to 1 month.

18. Centrifuge the glass tubes with the middle layer for 5 min at 2000× *g* at 0 °C and remove any potentially remaining polar and non-polar layers.
19. In the fume hood, add 600 µL of cold modified Buffer RLT to the middle layer left in the 8 mL glass tubes.

- When the tissue is not difficult to lyse and/or less than 20 mg of tissue has been prepared, add 350 µL of the modified Buffer RLT.

20. Vortex for 30 s at max speed.
21. In the fume hood, transfer the lysate on to one QIAshredder column placed in a 2 mL collection tube. Centrifuge the loaded QIAshredder column for 2 min at 16,100× *g* at room temperature.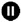. **PAUSE STEP:** Close the tube using the cap provided with the QIAshredder, and store the cell lysate at −80 °C until further analysis. Resume from step 42.

### 3.5. Lyophilization of the Polar Metabolite and NMR Analysis. Time for Completion: 2–3 Days

22. Centrifuge the collected polar layer sample at 10,000× *g* for 2 min and transfer the supernatant into a new 15 mL falcon tube to avoid potential contaminants such as the non-polar layer or cell debris. Measure and record the volume.
23. To the polar metabolite layer, add three parts of Type I water.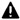. **CRITICAL STEP:** Make sure this final volume is not more than half of the tube volume. If it is, then transfer it to a larger tube; this helps prevent sample loss during the drying step.
24. Store at −80 °C for at least 12 h in an upright or slightly tilted position so that the sample solution does not reach the top of the tube, which could lead to sample loss during the next step
25. Use a MiVac Duo concentrator to evaporate the methanol and water solvent using the instrument’s suggested settings for this solvent mixture.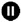. **PAUSE STEP:** Store the dried samples at −80 °C until further analysis.
26. For analysis using NMR spectroscopy, reconstitute the dried sample in 270 µL of 10 mM phosphate buffer (pH 6.85).
27. Centrifuge the sample for 15 min at 14,000× *g* at 4 °C, and mix 207 µL of the supernatant with 23 µL of DSS-d6 (internal standard).
28. Adjust the pH to approximately 6.8 for each sample as needed by adding a small volume of 1 N HCl or NaOH.
29. Transfer 180 µL of the sample into a 3 mm NMR tube and store at 4 °C until NMR data acquisition, which should be within 24 h of the sample preparation.
30. Acquire all ^1^H NMR spectra at 25 °C using the noesypr1d pulse sequence on a Bruker Avance 600 MHz NMR spectrometer as described [3].

### 3.6. Total Oxylipin (free and bound) Extraction. Time for Completion: 3:00 Hours per Batch of 12 Samples

31. Dry the collected non-polar chloroform extract under nitrogen and reconstitute with 1500 μL chloroform:isopropanol (2:1).
32. Dry 500 μL of the reconstituted extract under nitrogen gas and dissolve it in 200 μL of methanol containing 0.1% BHT and 0.1% acetic acid, 10 μL of antioxidant solution containing 0.2 mg/mL BHT, EDTA and triphenylphosphine (TPP) in water:methanol (1:1), and 10 µL of surrogate standard solution containing 2 μM of d11-11(12)-EpETrE, d11-14,15-DiHETrE, d4-6-keto-PGF1α, d4-9-HODE, d4-LTB4, d4-PGE2, d4-TXB2, d6-20-HETE and d8-5-HETE in methanol.
33. Add 200 μL of 26.5 mg/mL sodium carbonate dissolved in methanol:water (1:1) to the sample, and hydrolyze at 60 °C for 30 min.
34. After cooling down the sample, add 25 μL of acetic acid and 1575 μL of water.
35. Confirm that the pH is between 4 and 6 in one or more representative samples, by adding one drop of the mixture (from step 34) to litmus paper and observing the change in color.
36. Pour the sample into a 60 mg Waters Oasis HLB 3cc SPE column pre-rinsed with one volume of ethyl acetate and two volumes of methanol, and pre-conditioned with two volumes of SPE buffer containing 5% methanol and 0.1% acetic acid in Type I ultrapure water.
37. Rinse the column twice with SPE buffer before subjecting to 20 min of vacuum (≈20 psi).
38. Elute oxylipins with 0.5 mL methanol and 1.5 mL ethyl acetate and collect in a 2 mL centrifuge tube.
39. Dry the samples under nitrogen and reconstitute in 100 µL methanol.

### 3.7. Ultraperformance Liquid Chromatography-tandem Mass Spectrometer (UHPLC-MS/MS) Analysis. Time for Completion: 6:00 Hours per Batch of 12 Samples

40. Inject 10 µL of the sample into an Agilent LC system 1290, coupled to an Agilent 6460 Triple Quadrupole MS system (UPLC-MS/MS). The system is programmed to analyze 72 oxylipins species.
41. Separate oxylipin species on an Agilent Eclipse Plus C18 column (2.1 × 150 mm, 1.8 μm) at 45 °C with a binary gradient consisting of solvent A (water containing 0.1% acetic acid) and solvent B (acetonitrile:methanol (80:15 v:v) containing 0.1% acetic acid) (Table S1). Use electrospray ionization (negative mode) as the ion source with the experimental parameters as follows: gas temperature = 250 °C, gas flow = 10 L/min; sheath gas temperature = 300 °C; sheath gas flow = 11 mL/min; nebulizers = 35 psi; capillary voltage = 3500 V/−3500 V. The optimized mass-spectrometry parameters for measuring oxylipins are summarized in Table S2.

### 3.8. Cell Lysis Preparation for the Subsequent Extractions. Time for Completion: 0:15 Hours per Batch of 12 Samples

The remaining procedures on the cell lysate are done in the BSC to avoid contamination. The following steps were taken from the manual of the AllPrep DNA/RNA/Protein Mini Kit (step 4 on page 31 to step 24, as well as Appendix E1–E4 on page 52; December 2014 version), RNeasy MinElute Cleanup Kit (Supplementary protocol RY38; November 2008 version), as well as RNase-Free DNase Set (June 2018 version). The combined flow of the protocol is stated below with some modifications.

42. Incubate the frozen cell lysate in a heater set at 30 °C until thawed.
43. Centrifuge the lysate for 3 min at 14,000× *g* at room temperature, and transfer the supernatant to an AllPrep DNA spin column placed in a 2 mL collection tube.
44. Close the lid gently, centrifuge for 30 s at 8000× *g*.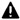. **CRITICAL STEP:** Up to 700 μL of sample can be loaded onto the AllPrep DNA spin column. Make sure that no liquid remains on the column membrane after centrifugation. If necessary, repeat the centrifugation until all liquid has passed through the membrane.
45. Transfer the AllPrep DNA spin column into a new 2 mL collection tube, and store at 4 °C for later DNA purification (starting at step 72).

### 3.9. Total RNA Purification. Time for Completion: 1:00 Hours per Batch of 12 Samples

46. To the flow-through from step 45, add 430 μL of 96–100% ethanol. Mix well using a pipette by drawing the liquid up and down.
47. To the RNeasy spin column in a 2 mL tube, transfer a maximum of 700 μL of sample onto the top.
48. Close the lid of each column, place into the centrifuge, and centrifuge for 15 s at 8000× *g*.
49. Transfer the flow-through to a 2 mL tube for small RNA and protein precipitation (step 60).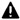. **CRITICAL STEP:** If the sample volume is more than 700 μL, repeat the process until the entire sample has passed through the same RNeasy spin column. Collect the flow-through after each centrifugation to the same 2 mL tube.
50. Wash the RNeasy spin column by pipetting 350 μL of Buffer RW1 onto the RNeasy spin column and centrifuge for 15 s at 8000× *g*.

- Buffer RW1 contains guanidine thiocyanate, which will help to prevent any RNase activity and help to remove carbohydrates, fatty acids, proteins, and other biomolecules.

51. Mix 10 μL of DNase I stock solution and 70 μL of Buffer RDD (supplied from the RNase-free DNase Set). Gently invert the tube to mix, and centrifuge briefly to collect the solution to the bottom of the tube.

- Buffer RDD is used to prepare the DNase solution.

52. Add 80 μL of DNase I incubation mix from step 51 directly onto the RNeasy spin column membrane, and incubate for 15 min at room temperature.
53. Wash the RNeasy spin column again by adding 350 μL of Buffer RW1 to the RNeasy spin column and close the lid of each column. Centrifuge for 15 s at 8000× *g* and discard the flow-through.
54. Wash the spin column a third time by adding 500 μL of Buffer RPE. Close the lid of each spin column and centrifuge for 15 s at 8000× *g*. Discard the flow-through.

- Buffer RPE needs to be diluted with ethanol as instructed before use. Use to wash the spin column membrane to remove traces of salt.

55. Add another 500 μL of Buffer RPE to the RNeasy spin column and close the lid. Centrifuge for 2 min at 8000× *g* and discard the flow-through.
56. Place the RNeasy spin column in a new 2 mL tube. Centrifuge at 16,100× *g* for 1 min.
57. Place the RNeasy spin column in a new 1.5 mL tube and add 50 μL of RNase-free water directly to the spin column membrane.
58. Close the spin column lid, and centrifuge for 1 min at 8000× *g* to elute the RNA.
59. Aliquot 1 μL of the flow-through for a quality check via Nanodrop, 4 μL for analysis via the Bioanalyzer (using an Agilent RNA 6000 Nano Kit to prepare the sample), and 3 μL for quantification by Qubit (using the Qubit RNA HS Assay Kit to prepare the sample). The rest should be saved for mRNA analysis. Store samples at −80 °C.

- RNA samples can be stored at −80 °C for 1 year.

### 3.10. Small RNA Purification. Time for Completion: 1:10 Hours per Batch of 12 Samples

60. Add 600 μL of Buffer APP to the flow-through from step 49. Vortex for 5 s and incubate at room temperature for 10 min.

- Buffer APP contains zinc chloride to precipitate protein.

61. Centrifuge at 16,100× *g* for 10 min, and carefully transfer the supernatant to a new 5 mL tube. While transferring, measure the approximate volume (needed for the next step). Keep the pellet for total protein precipitation at room temperature (Step 69).
62. Add 1 volume of 100% ethanol to the supernatant from step 61, and mix well using a pipette by drawing the liquid up and down.
63. To the RNeasy MinElute spin column in a 2 mL tube (supplied in the RNeasy MinElute Cleanup Kit), transfer a maximum of 700 μL of the sample. Close the lid on each tube, and centrifuge for 15 s at 8000× *g*. Discard the flow-through and repeat until the entire sample has passed through the RNeasy MinElute membrane.
64. Place the RNeasy MinElute spin column in a new 2 mL tube. Add 500 μL of Buffer RPE to the spin column and close the lid. Centrifuge for 15 s at 8000× *g* and discard the flow-through.
65. Add 500 μL of 80% ethanol to the RNeasy MinElute spin column and close the lid. Centrifuge for 2 min at 8000× *g* and discard the flow-through and carefully place the RNeasy MinElute spin column into a new 2 mL collection tube.
66. Centrifuge at 16,100× *g* for 5 min keeping the lid of the spin column open to allow it to dry completely.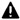. **CRITICAL STEP:** To avoid damage to the lids, place the tubes with spin columns into the centrifuge with at least one empty position between them and orient the lids to point in a direction opposite to the rotation of the rotor.
67. Place the RNeasy MinElute spin column in a new 1.5 mL tube. Pipette 14 μL RNase-free water directly to the center of the spin column membrane and close the lid. Centrifuge for 1 min at 16,100× *g* to elute the small RNA.
68. Aliquot 1 μL of the flow-through for a quality check via Nanodrop and 3 μL for quantification by Qubit (using the Qubit microRNA Assay Kit for sample preparation). The remaining sample should be saved for small RNA analysis. Store the samples at −80 °C.

### 3.11. Total Protein Precipitation. Time for Completion: 0:15 Hours per Batch of 12 Samples

69. To the protein pellet obtained in step 61, add 500 μL of 70% ethanol.
70. Centrifuge at 16,100× *g* for 1 min, and remove the supernatant using a pipette, removing as much liquid as possible.
71. Dry the protein pellet for 10 min at room temperature until the pellet is completely dried.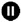. **PAUSE STEP:** Store the protein pellet at −80 °C.

### 3.12. DNA Purification. Time for Completion: 0:20 Hours per Batch of 12 Samples

72. Aliquot the required amount of Buffer EB and preheat to 70 °C to ensure optimal DNA elution.

- Buffer EB is used to elute DNA.

73. Add 500 μL of Buffer AW1 to the AllPrep DNA spin column from step 45 and close the lid of the tube. Centrifuge for 15 s at 8000× *g* and discard the flow-through.

- Buffer AW1 contains guanidine hydrochloride used to wash the spin column membrane. Buffer AW1 needs to be diluted with ethanol before use.

74. Pipette 500 μL of Buffer AW2 to the AllPrep DNA spin column and close the tube lid. Centrifuge for 2 min at 16,100× *g*.

- Buffer AW2 is used to wash the column. Buffer AW2 needs to be diluted with ethanol before use.

75. Carefully place the AllPrep DNA spin column in a new 1.5 mL tube.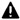. **CRITICAL STEP:** If the column contacts the flow-through, centrifuge the spin column again for 1 min at 16,100× *g* after emptying the collection tube.
76. Add 100 μL Buffer EB preheated to 70 °C directly to the spin column membrane, close the lid, and incubate at room temperature for 2 min. Centrifuge for 1 min at 8000× *g* to elute the DNA.
77. Aliquot 1 μL of the flow-through for a quality check via Nanodrop and 3 μL for quantification by Qubit using Qubit dsDNA BR Assay Kit. The rest should be saved for the DNA analysis. Store the samples at −80 °C.

### 3.13. Protein Reconstitution and Quantification. Time for Completion: 1:10 Hours per Batch of 12 Samples

78. Set a heat block and benchtop centrifuge at 37 °C.
79. Add 100 µL of 5% w/v SDS into each tube with protein pellet from step 71.
80. Using a 100 μL pipette tip, physically poke to disturb the pellet into pieces.
81. Vortex the sample at high speed for 1 min.
82. Incubate the sample at 37 °C for 30 min.
83. Vortex the sample at high speed for 5 min.
84. If large pellets still remain at the bottom of the tubes, repeat incubation (15 min at 37 °C) and vortex for 5 min.
85. Centrifuge the sample at 14,000× *g* at 37 °C for 1 min.
86. Transfer the supernatant to a new tube.
87. Quantify the protein using the DC Protein assay kit with Synergy H1 Hybrid multi-mode reader.

### 3.14. Further Protein Purification. Time for Completion: 0:20 Hours per Batch of 12 Samples

If the protein fraction obtained from this protocol does not give the desired result by Western blot, the following steps can be applied to desalt the protein sample. This protocol was adapted from the manufacturer instructions supplied with the Zeba Spin desalting columns, 7K MWCO (Thermo Fisher Scientific).

88. Dilute the reconstituted protein sample to 5–10 mg/mL.
89. Add 100 µL of the diluted protein sample to a Zeba Spin desalting column (0.5 mL) following the manufacturer’s instructions.
90. If the volume of the protein sample is <70 µL, 15 μL of Type I water should be loaded as the stacker after the protein solution is fully absorbed to the resin bed.
91. Centrifuge at 1500× *g* for 2 min to collect the desalted sample.

## 4. Results

**Figure 2:**
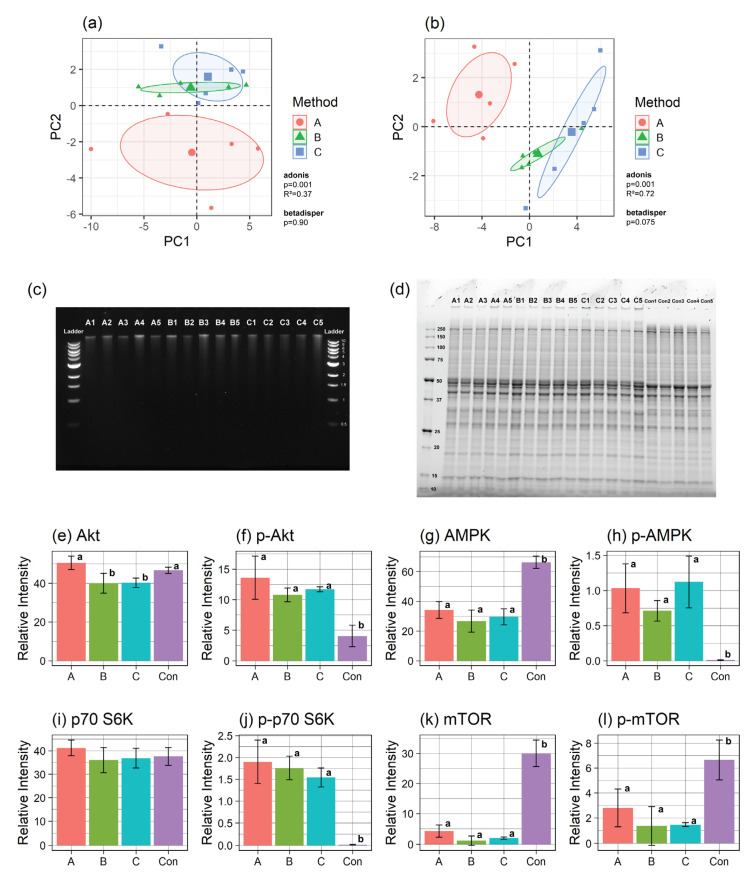
Validation of the extraction methods. PCA plots of the (**a**) polar and (**b**) non-polar metabolomics datasets. Ellipses indicate the 95% confidence interval, and the three larger shapes at the center of each ellipse indicate the centroids of the corresponding metabolite extraction method (Method A (red), Method B (green), Method C (blue). The statistical results from adonis (testing the centroids) and betadisper (testing the data dispersion) tests are included in each plot. (**c**) The result of DNA gel electrophoresis. The molecular weights of the DNA ladder are indicated in kb. (**d**) Result of protein SDS-PAGE. The molecular weights of the protein ladder are indicated in kDa. Bar plots of the relative intensity of total and phospho-protein of Akt (**e**,**f**), AMPK (**g**,**h**), p70 S6K (**i**,**j**), and mTOR (**k**,**l**), respectively. Different superscript letters indicate significant differences between the groups found by the one-way ANOVA and post-hoc test after multiple comparison testing. Error bars indicate SD of five replicates per extraction method except for Con in (**k**) and (**l**), which were generated from two replicates. For protein analysis, control protein samples were prepared by extracting protein from the same cryoground brain tissue using RIPA extraction buffer. Abbreviations: A1–A5, replicates of Method A; B1–B5, replicates of Method B; C1–C5, replicates of Method C; Con1–Con5 and Con, replicates of control protein sample.

A total of 43 polar metabolites were identified and quantified using ^1^H NMR (Table S3). The overall profile was visualized using principal component analysis (PCA) (Figure 2a), revealing the centroid of Method A significantly different (*p* = 0.001) from the centroids of Methods B and C. One-way analysis of variance (ANOVA) revealed a significantly higher level of ascorbate (*p* < 0.01) extracted by Method A (Table S3), whereas the other 42 metabolites were not different. To assess the variability within each method, the coefficient of variation (CV) of the five technical replicates was calculated for each metabolite and plotted as a density plot (Figure S1a). Metabolites extracted with Methods B and C had lower mean CVs than those extracted with Method A.

Thirty species of total oxylipins (i.e., both free and esterified) were found by UHPLC-MS/MS (Table S4). The overall profile was visualized by PCA (Figure 2b), revealing that Method A was different from Methods B and C (*p* = 0.001). One-way ANOVA showed that 14 out of 30 oxylipin species had significantly different total yields between the methods, with Method A having higher concentrations for most oxylipins (Table S4). A density plot generated using the CVs of the total yield revealed that Method B had the lowest CV, while Method A had the highest (Figure S1b). To assess the loss in the surrogate deuterated internal standards during the hydrolysis, the concentration of the surrogate measured after hydrolysis was divided by the concentration originally added to the sample to calculate percent recovery (Table S5). The percent recovery from Method A was lower than from Methods B and C, with statistical significance found for d-11-11(12)EpEtrE and d11-14,15-DiHETrE (*p* = 0.030 for both surrogates), suggesting that the high total yields found in Method A were an artifact caused by the low recovery of the surrogate standard. It is likely that the addition of antioxidants BHT and EDTA in Methods B and C, as well as the extra extraction step, resulted in better performance. The addition of KCl in Method C did not impact the determination of polar metabolite or total oxylipin concentrations.

The AllPrep DNA/RNA/Protein Mini Kit was used to extract genomic DNA, RNA longer than 200 nucleotides (nt), and protein, and the RNeasy MinElute Cleanup Kit was utilized to isolate small RNA. After extraction, quantities of DNA, RNA, and small RNA were determined for each method by Qubit Fluorometer, with total yields of 1.24–1.95 mg of DNA, 3.38–9.18 μg of RNA, and 0.27–0.48 μg of small RNA (Table S6). Nanodrop spectrophotometer 260/280 measurements were above 1.9 for DNA and above 2.0 for longer RNA for all methods (Table S7) (values of 1.8 or 2.0 are considered good for DNA or RNA, respectively). The quality of DNA was further assessed via agarose gel electrophoresis (Figure 2c), where all samples showed clear single bands with molecular weights above 10 kb. RNA is susceptible to degradation by RNase that is abundant and ubiquitous [4], and thus its quality needs to be assessed via Bioanalyzer prior to downstream analyses. All three methods showed an average RNA Integrity Number (RIN) above 7.0 (Table S7). It has been reported that RNA is considered to be pure and acceptable for downstream analyses if the RIN > 7 [5]. Although a few samples from Methods B and C showed RIN values between 6.2 and 6.9, previous publications found that RNA can be adequately sequenced with RIN as low as 4 [6]. While all three multi-extraction methods yielded a sufficient quantity of DNA, RNA, and small RNA for downstream analyses, the addition of KCl in Method C caused the middle layer to aggregate, making it easier to collect the polar and non-polar layers while minimizing disruption of the middle layer. Considering that Method B resulted in lower RNA concentration (approximately half of Methods A and C) as well as a large variability, Method C is preferred for RNA isolation.

The flow-through collected after RNA binding to the RNeasy spin column was incubated with Buffer APP to precipitate protein. The total protein extracted using the multi-extraction method was compared with protein extracted using a conventional method (Control) [7]. All multi-extraction methods yielded enough total protein for downstream analyses (1.85–2.35 mg) (Table S8). The quality of the extracted protein was first assessed by sodium dodecyl sulphate-polyacrylamide gel electrophoresis (SDS-PAGE) (Figure 2d), which showed clear bands that were well separated in all samples. Except for a band just above 250 kDa observed for samples extracted using the conventional method, the protein bands showed similar patterns for Methods A-C. The quality of protein was further assessed by Western blot on selected proteins of the mTOR pathway (Akt, AMPK, and p70 S6K) because these proteins are of various molecular weights and can be phosphorylated, allowing us to determine whether this method was capable of assessing levels of phosphorylated proteins as well. All three multi-extraction methods were able to detect the total and phospho-proteins of AMPK and p70 S6K at similar relative intensity, except for total Akt (Method A was significantly higher than B and C) (Figure 2e–j, Figure S2a). All multi-extraction methods were significantly more effective in extracting phosphoproteins than the conventional method.

In protein samples collected as part of the multi-extraction procedures, mTOR and phospho-mTOR (p-mTOR) were not detected (Methods B and C) or had very faint expression (Method A), whereas a strong and clear band was detected from protein extracted by the conventional method (Figure S2b). This might be due to residual guanidinium salt used for the extraction of nucleic acids, since guanidine thiocyanate changes protein structure [8]. Consequently, we desalted the protein fractions using a Zeba Spin Desalting Column. Although little difference between before and after the desalting treatment could be observed on the SDS-PAGE gel band image (Figure S2c), it allowed us to capture clear mTOR and p-mTOR bands in all samples extracted using the multi-extraction methods (Figure 2k,l and Figure S2d), with Method C showing the most consistency amongst the multi-extraction methods. To assess the impact of desalting on proteins other than mTOR and p-mTOR, after application of Method C, proteins were extracted in triplicate, and expression of both Akt and p-Akt was compared. Although a protein loss of ~20% was observed after desalting, more consistent bands of Akt and p-Akt were observed (Figure S2e). Notably, the relative intensities of Akt or p-Akt did not statistically differ between pre- and post-desalting (Figure S2f,g; *p* = 0.42 and 0.73 by t-test for Akt and p-Akt, respectively).

In conclusion, we determined that Method C provides superior results for the simultaneous extraction of metabolites, DNA, RNA, small RNA, and protein from a small tissue sample. Substantial improvements over the previous method [1,9] for the accurate measurement of both polar and non-polar metabolites (oxylipins), better reproducibility, as well as additional steps to improve downstream recovery of protein were made. Our improved method will provide sufficient quantity and quality of biomolecules for downstream analyses for any application.

## Supplementary Materials

The following are available online at https://www.mdpi.com/2409-9279/3/3/61/s1, Figure S1: Density plots of the coefficient of variation (CV) calculated for polar metabolites and non-polar metabolites, Figure S2: Comparison of protein recovery by Western blot before and after desalting, Table S1: Gradient conditions for oxylipin separation via LC-MS/MS analysis, Table S2: Optimized mass-spectrometry parameters for measuring oxylipins, Table S3: Concentration of polar metabolites, Table S4: Total yield of oxylipin, Table S5: Percent recovery of oxylipin surrogates, Table S6: The concentration of DNA, RNA, and small RNA measured by Qubit, Table S7: Summary of quality assessments for DNA and RNA, Table S8: The yield of protein and the total yield of brain tissue after analysis by the DC Protein Assay kit, Table S9: A list of abbreviations of oxylipin species and their internal standards.
