# Supplementary material for: Optimization of a Method for the Simultaneous Extraction of Polar and Non-Polar Oxylipin Metabolites, DNA, RNA, Small RNA, and Protein from a Single Small Tissue Sample
Source: Methods Protoc. 2020 Aug 26;3(3):61. doi: 10.3390/mps3030061 (PMC7564281; doi:10.3390/mps3030061)
Supplement: Supplementary file 1 [file mps-03-00061-s001.pdf]

Supplementary Information

Optimization of a method for the simultaneous extraction of polar and non-polar oxylipin metabolites, DNA, RNA, small RNA, and protein from a single small tissue sample

Yu Hasegawa, Yurika Otoki, Shannon McClorry, Laurynne C. Coates, Rachel L. Lombardi, Ameer Y. Taha, Carolyn M. Slupsky

Procedure for Methods A and B

For Methods A and B, the following steps were modified:

3.1. Preparation of Reagents

1. Prepare chloroform:methanol (2:1) with 0.002% BHT [*Solution 1*]. Pre-chill in a -20 °C freezer.
2. Prepare 1 mM EDTA dissolved in Type I water [*Solution 2*]. Pre-chill to 4 °C.
3. Prepare chloroform:methanol (10:1) [*Solution 3*]. Pre-chill in a -20 °C freezer.

3.3. Metabolite Extraction

3.3.1. Method A

1. Add 1600 μL of chloroform, 800 μL of methanol, and 600 μL of Type I ultrapure water to tubes with ground brain tissue and mix by vortexing for 20 seconds.
2. Centrifuge the tubes for 15 min at 2,000 rpm at 0 °C to separate the sample into three layers.
3. Collect the upper layer into a new 15 mL conical centrifuge tube. Do not disturb the cell layer. Keep the tube on ice and proceed to step 22.
4. Using a 9-inch glass Pasteur pipette, collect the bottom layer and place in a new 8 mL glass tube. Place the tube on ice and proceed to step 31.
5. Proceed to step 42 to process the middle layer.

3.3.2. Method B

1. Add 2.4 mL of cold *Solution 1* into the 8 mL glass tube with the cryoground tissue.
2. Add 600 μL of *Solution 2*.
3. Cap carefully and vortex for 20 s at max speed, and centrifuge for 15 min at 2,000 rpm at 0 °C.
4. Using a 9-inch glass Pasteur pipette, collect the bottom layer and place in a new 8 mL glass tube. Place the tube on ice.
5. Add 1.4 mL of cold *Solution 3* to the remaining upper and cell layer.
6. Vortex for 10 s at max speed, and centrifuge for 15 min at 2,000 rpm at 0 °C.
7. Collect the upper layer into a new 15 mL conical centrifuge tube. Do not disturb the cell layer. Keep the tube on ice and proceed to step 22.
8. Collect the bottom layer with a Pasteur pipette, and add to the glass tube from step 4. Place the tube on ice and proceed to step 31.
9. Proceed to step 42 to process the middle layer.

Supplementary Tables

Table S1. Gradient conditions for oxylipin separation via LC-MS/MS analysis. Solvent B consisted of acetonitrile:methanol (80:15) containing 0.1% acetic acid.

Time (min)	Solvent B concentration (%)	Flow (min/mL)
0	35	0.3
3	40	0.25
4	48	0.25
10	60	0.25
20	70	0.25
24	85	0.25
24.5	85	0.25
24.6	100	0.35
26	100	0.35
26.1	35	0.35
27.3	35	0.3
28	Stop	

Table S2. Optimized mass-spectrometry parameters for measuring oxylipins.

Compound name	Precursor Ion (m/z)	Product Ion (m/z)	Frag (V)	CE (V)	Cell Acc (V)	Rt (min)	Internal standard
20-COOH-LTB4	365.2	347.2	120	7	4	4.0	d4-LTB4
Resolvin E1	349.3	195	115	10	4	4.6	d4-PGE2
d4-6-keto-PGF1a	373.3	167.1	90	19	4	4.7	-
6-keto-PGF1a	369.3	163.2	90	22	4	4.7	d4-6-keto-PGF1a
20-OH-LTB4	351.2	195.2	95	13	4	5.0	d4-LTB4
d4-TXB2	373.3	173.2	105	10	4	6.1	-
TXB2	369.2	169.1	80	10	4	6.1	d4-TXB2
PGE3	349.3	269.2	120	7	4	6.2	d4-PGE2
PGD3	349.3	269.2	120	7	4	6.5	d4-PGE2
9,12,13-TriHOME	329.2	211.1	125	16	4	6.6	d4-PGE2
9,10,13-TriHOME	329.2	171.1	110	16	4	6.7	d4-PGE2
PGF2a	353.2	309.2	120	10	4	6.7	d4-PGE2
d4-PGE2	355.2	275.3	90	7	4	7.0	-
PGE2	351.2	271.3	80	10	4	7.0	d4-PGE2
PGD1	353.3	317.2	75	7	4	7.2	d4-PGE2
PGE1	353.3	317.2	75	7	4	7.3	d4-PGE2
PGD2	351.2	271.3	80	10	4	7.3	d4-PGE2
LTD4	495.3	177.1	55	13	4	7.8	d4-LTB4
LXA4	351.2	115.2	95	10	4	8.1	d4-LTB4
LTC4	624.3	272.1	70	22	4	9.6	d4-LTB4
LTE4	438.2	333.3	90	13	4	9.7	d4-LTB4
PGJ2	333.3	189.2	90	10	4	9.9	d4-PGE2
PGB2	333.3	175.1	125	13	4	10.0	d4-PGE2
6-trans-LTB4	335.2	195.1	125	7	4	10.8	d4-LTB4
5,15-DiHETE	335.2	173.2	95	7	4	10.8	d11-14,15-DiHETrE
5,6-DiHETE	335.2	115.2	90	4	4	10.9	d11-14,15-DiHETrE
8,15-DiHETE	335.2	235.2	90	7	4	11.1	d11-14,15-DiHETrE
17,18-DiHETE	335.3	247.2	105	7	4	11.7	d11-14,15-DiHETrE
d4-LTB4	339.2	197.2	80	10	4	11.9	-
LTB4	335.2	195.1	125	7	4	11.9	d4-LTB4

14,15-DiHETE	335.3	207.2	95	7	4	12.3	d11-14,15-DiHETrE
9,10-DiHOME	313.2	201.2	130	16	4	12.42	d11-14,15-DiHETrE
12,13-DiHOME	313.2	183.2	130	16	4	12.7	d11-14,15-DiHETrE
14,15-DiHETrE	337.2	207.1	130	10	4	13.8	d11-14,15-DiHETrE
d11-14,15-DiHETrE	348.2	207.1	125	10	6	13.8	-
LTB3	337.2	195.2	120	7	4	14.2	d4-LTB4
11,12-DiHETrE	337.2	167.1	120	13	4	14.8	d11-14,15-DiHETrE
9-HOTrE	293.2	171.2	110	4	4	15.3	d4-9HODE
8,9-DiHETrE	337.2	127.1	85	13	4	15.6	d11-14,15-DiHETrE
13-HOTrE	293.2	195.1	125	10	4	15.6	d4-9HODE
15-deoxy-PGJ2	315.2	271.2	130	4	4	16.1	d4-PGE2
d6-20-HETE	325.2	281.2	110	7	4	16.5	-
20-HETE	319.2	275.1	125	10	4	16.5	d6-20-HETE
15-HEPE	317.2	219.2	90	4	4	16.6	d8-5-HETE
5,6-DiHETrE	337.2	145.1	85	7	4	16.7	d11-14,15-DiHETrE
8-HEPE	317.2	155.2	115	7	4	17.0	d8-5-HETE
12-HEPE	317.2	179.2	110	4	4	17.2	d8-5-HETE
5-HEPE	317.2	115.1	115	4	4	17.8	d8-5-HETE
d4-9HODE	299.2	172.3	90	13	4	18.0	-
13-HODE	295.2	195.2	95	13	4	18.0	d4-9HODE
9-HODE	295.2	171.1	120	10	4	18.0	d4-9HODE
15-HETE	319.2	219.2	120	4	4	19.1	d8-5-HETE
17(18)-EpETE	317.2	215.2	130	4	4	19.2	d-11-11(12)EpEtrE
13-oxo-ODE	293.2	195.1	95	13	4	19.3	d6-20-HETE
17-HDoHE	343.2	281.2	95	4	4	19.5	d4-9HODE
11-HETE	319.2	167.2	100	7	4	19.5	d8-5-HETE
9-HETE	319.2	167.2	80	7	4	19.8	d8-5-HETE
9-oxo-ODE	293.2	185.1	90	13	4	20.0	d6-20-HETE
15-oxo-ETE	317.2	113.1	125	10	6	20.1	d8-5-HETE
14(15)-EpETE	317.2	207.2	100	4	4	20.2	d-11-11(12)EpEtrE
8-HETE	319.2	155.2	120	7	4	20.4	d8-5-HETE
11(12)-EpETE	317.2	167.2	90	4	4	20.5	d-11-11(12)EpEtrE
12-HETE	319.2	179.2	120	7	4	20.5	d8-5-HETE

8(9)-EpETE	317.2	127.2	115	4	4	20.8	d-11-11(12)EpEtrE
12-oxo-ETE	317.2	153.1	115	7	4	21.1	d8-5-HETE
15(S)-HETrE	321.2	221.2	85	7	4	21.1	d8-5-HETE
5-HETE	319.2	115.1	90	10	4	21.2	d8-5-HETE
d8-5-HETE	327.2	116.1	75	7	4	21.2	-
12(13)EpOME	295.3	195.2	95	7	4	22.5	d-11-11(12)EpEtrE
19(20)-EpDPE	343.2	241.2	130	7	4	22.5	d-11-11(12)EpEtrE
14(15)-EpETrE	319.2	219.3	130	4	4	22.8	d-11-11(12)EpEtrE
9(10)-EpOME	295.3	171.1	100	7	4	22.8	d-11-11(12)EpEtrE
16(17)-EpDPE	343.2	233.2	130	4	4	23.3	d-11-11(12)EpEtrE
13(14)-EpDPE	343.2	193.2	80	4	4	23.4	d-11-11(12)EpEtrE
5-oxo-ETE	317.2	273.2	120	7	4	23.4	d8-5-HETE
11(12)-EpETrE	319.2	167.2	105	4	4	23.5	d-11-11(12)EpEtrE
d-11-11(12)EpEtrE	330.2	167.2	80	7	4	23.5	-
10(11)-EpDPE	343.2	153.2	90	4	4	23.6	d-11-11(12)EpEtrE
7(8)-EpDPE	343.2	113.1	85	4	4	23.8	d-11-11(12)EpEtrE
8(9)-EpETrE	319.2	167.2	90	4	4	23.9	d-11-11(12)EpEtrE
5(6)-EpETrE	319.2	191.1	115	4	4	24.2	d-11-11(12)EpEtrE

Abbr: m/z, mass to charge ratio; CE, collision energy; Rt, Retention Time.

Table S3. Concentration of polar metabolites (nmol/mg)*.

	Method A	Method B	Method C	p-value**
4-Aminobutyrate	851 ± 195	795 ± 86	787 ± 117	NS
AMP	843 ± 167	1011 ± 173	1060 ± 69	NS
Acetate	366 ± 176	167 ± 78	133 ± 61	NS
Alanine	452 ± 75	439 ± 49	462 ± 56	NS
Ascorbate	545 ± 98 [a]	65 ± 16 [b]	59 ± 41 [b]	<0.001
Aspartate	1249 ± 237	1251 ± 178	1336 ± 138	NS
Betaine	58 ± 10	56 ± 6	58 ± 6	NS
Choline	58 ± 17	42 ± 5	54 ± 8	NS
Creatine	3991 ± 745	3947 ± 517	4071 ± 414	NS
Dimethyl-sulfone	8 ± 3	9 ± 4	13 ± 5	NS
Ethanolamine	133 ± 40	83 ± 51	103 ± 44	NS
Formate	291 ± 187	381 ± 123	328 ± 104	NS
Fumarate	30 ± 6	33 ± 10	25 ± 14	NS
Glutamate	5651 ± 1071	5681 ± 810	6052 ± 679	NS
Glutamine	6347 ± 902	6396 ± 918	6415 ± 594	NS
Glutathione	526 ± 117	490 ± 102	573 ± 109	NS
Glycerol	188 ± 46	192 ± 27	191 ± 39	NS
Glycine	354 ± 60	359 ± 38	364 ± 38	NS
Guanosine	41 ± 14	29 ± 8	34 ± 8	NS
Hypoxanthine	79 ± 33	80 ± 19	92 ± 22	NS
IMP	44 ± 14	49 ± 15	41 ± 18	NS
Inosine	446 ± 199	220 ± 28	225 ± 67	NS
Isoleucine	85 ± 19	73 ± 16	92 ± 5	NS
Lactate	10843 ± 2228	11237 ± 1646	11554 ± 1181	NS
Leucine	81 ± 22	87 ± 14	103 ± 22	NS
Lysine	223 ± 64	212 ± 45	183 ± 44	NS
N-Acetylaspartate	5547 ± 1361	5836 ± 886	6154 ± 616	NS
N-Acetylglutamate	58 ± 16	55 ± 26	47 ± 14	NS
NAD+	115 ± 72	179 ± 58	148 ± 25	NS
O-Phosphocholine	191 ± 35	238 ± 38	194 ± 21	NS
O-Phosphoethanolamine	1580 ± 349	1625 ± 248	1458 ± 120	NS
Ornithine	55 ± 17	49 ± 9	88 ± 35	NS
Propylene-glycol	109 ± 55	189 ± 125	174 ± 99	NS
Serine	330 ± 98	300 ± 41	281 ± 73	NS
Succinate	302 ± 58	295 ± 44	303 ± 19	NS
Taurine	443 ± 135	426 ± 60	505 ± 84	NS
Threonine	723 ± 163	657 ± 101	756 ± 99	NS
Tyrosine	171 ± 37	153 ± 36	155 ± 4	NS
Uridine	55 ± 18	49 ± 10	39 ± 19	NS
Valine	112 ± 14	111 ± 11	124 ± 14	NS
myo-Inositol	1967 ± 353	1914 ± 222	2072 ± 212	NS

sn-Glycero-3-phosphocholine	318	±	60	270	±	70	389	±	41	NS
β-Alanine	43	±	13	46	±	13	38	±	15	NS

*Data are mean ± SD of five replicates per extraction method.

**Data were analyzed by one-way ANOVA (p-values were generated after false discovery rate (FDR) correction) followed by Tukey's post-hoc test. Different superscript letters indicate significant differences between groups.

Abbreviations: AMP, adenosine monophosphate; IMP, inosine monophosphate; NAD, nicotinamide adenine dinucleotide; NS, not significant.

Table S4. Total yield of oxylipin (pmol/mg)*.

	Method A				Method B				Method C				p-value**
10(11)-EpDPE	0.69	±	0.23	a	0.44	±	0.057	b	0.45	±	0.060	b	0.050
11(12)-EpETE	0.016	±	0.015		0.021	±	0.0060		0.011	±	0.010		NS
11(12)-EpETrE	1.71	±	0.89	a	0.50	±	0.16	b	0.51	±	0.059	b	0.012
12(13)EpOME	1.91	±	0.81	a	0.69	±	0.15	b	0.59	±	0.065	b	0.0070
13(14)-EpDPE	5.059	±	1.27	a	4.01	±	0.56	ab	2.65	±	0.39	b	0.0083
14(15)-EpETrE	22.73	±	7.80	a	13.84	±	2.33	b	8.09	±	1.02	b	0.0070
16(17)-EpDPE	7.072	±	1.82	a	4.96	±	0.77	b	2.99	±	0.37	c	0.0052
19(20)-EpDPE	12.14	±	4.64	a	4.40	±	1.38	b	3.12	±	0.34	b	0.0052
5(6)-EpETrE	6.60	±	2.063	a	4.43	±	0.54	b	3.38	±	0.58	b	0.014
7(8)-EpDPE	3.49	±	0.96	a	2.13	±	0.35	b	1.55	±	0.18	b	0.0057
8(9)-EpETrE	3.52	±	1.089	a	2.41	±	0.35	ab	1.73	±	0.28	b	0.012
9(10)-EpOME	1.19	±	0.35	a	0.84	±	0.072	ab	0.56	±	0.099	b	0.0083
12,13-DiHOME	0.011	±	0.0050		0.0060	±	0.0010		0.0080	±	0.0010		0.084
14,15-DiHETrE	0.0050	±	0.0040		0.0070	±	0.0010		0.0070	±	0.0010		NS
5,6-DiHETE	0.024	±	0.014		0.011	±	0.0060		0.011	±	0.010		NS
5,6-DiHETrE	0.080	±	0.019	a	0.028	±	0.0050	b	0.020	±	0.0060	b	0.00025
13-HODE	2.84	±	0.39		3.11	±	0.61		3.46	±	0.91		NS
17-HDoHE	0.057	±	0.0080		0.067	±	0.013		0.060	±	0.012		NS
9-HODE	0.55	±	0.042		0.57	±	0.12		0.60	±	0.18		NS
13-oxo-ODE	0.030	±	0.0080		0.032	±	0.0080		0.037	±	0.0060		NS
9-oxo-ODE	0.031	±	0.0030		0.035	±	0.0080		0.035	±	0.0070		NS
11-HETE	0.066	±	0.011		0.055	±	0.0090		0.050	±	0.018		NS
12-HETE	0.058	±	0.0090		0.051	±	0.0080		0.044	±	0.014		NS
15-HEPE	0.14	±	0.033	a	0.12	±	0.016	ab	0.085	±	0.021	b	0.039
15-HETE	0.18	±	0.022		0.15	±	0.024		0.14	±	0.051		NS
15-oxo-ETE	0.017	±	0.0030		0.013	±	0.0030		0.015	±	0.0040		NS
15(S)-HETrE	0.0080	±	0.0030		0.0070	±	0.0010		0.0070	±	0.0020		NS
5-HETE	0.10	±	0.0030	a	0.079	±	0.013	b	0.071	±	0.018	b	0.012
8-HETE	0.055	±	0.010		0.040	±	0.0050		0.040	±	0.013		NS
9-HETE	0.046	±	0.0060		0.037	±	0.0060		0.036	±	0.0070		NS

*Data are mean ± SD of five replicates per extraction method.

**Data were analyzed by one-way ANOVA (p-values were generated after FDR correction) followed by Tukey's post-hoc test. Different superscript letters indicate significant differences between groups.

Abbreviations: NS, not significant

Table S5. Percent recovery of oxylipin surrogates*.

	Method A				Method B				Method C				p-value**
d-11-11(12)EpEtrE	14.80	±	2.61	a	22.59	±	3.82	b	26.60	±	5.24	b	0.030
d11-14,15-DiHETrE	58.89	±	12.89	a	86.74	±	8.49	b	80.17	±	8.76	b	0.030
d4-6-keto-PGF1a	63.24	±	8.71		68.17	±	5.82		62.76	±	6.37		NS
d4-9HODE	75.36	±	12.02		90.84	±	12.37		79.98	±	8.50		NS
d4-LTB4	50.13	±	9.03		50.14	±	8.63		57.61	±	7.29		NS
d4-PGE2	0.36	±	0.82		0.60	±	0.86		0.72	±	0.48		NS
d4-TXB2	55.09	±	8.88		61.09	±	6.52		56.64	±	6.16		NS
d6-20-HETE	77.21	±	9.82		92.33	±	7.97		82.11	±	5.21		NS
d8-5-HETE	62.88	±	11.61		79.40	±	8.51		70.88	±	8.11		NS

*Data are mean ± SD of five replicates per extraction method.
**Data were analyzed by one-way ANOVA (p-values were generated after FDR correction) followed by Tukey's post-hoc test. Different superscript letters indicate significant differences between groups.
Abbreviations: NS, not significant.

Table S6. The concentration of DNA (μg/μL), RNA (ng/μL), and small RNA (ng/μL) measured by Qubit.

	DNA			RNA			Small RNA		
	**Mean* (μg/μL)**	**CV (%)**	**Total yield** (mg)**	**Mean* (ng/μL)**	**CV (%)**	**Total yield** (μg)**	**Mean* (ng/μL)**	**CV (%)**	**Total yield*[illegible] (μg)**
Method A	12.38 ± 3.55	28.70	1.24 ± 0.36	183.52 ± 57.77	31.48	9.18 ± 2.89	19.61 ± 4.01	20.44	0.27 ± 0.056[illegible]
Method B	19.49 ± 6.97	35.75	1.95 ± 0.70	67.53 ± 50.14	74.25	3.38 ± 2.51	25.59 ± 18.03	70.27	0.36 ± 0.25[illegible]
Method C	19.48 ± 6.11	31.36	1.95 ± 0.61	117.82 ± 76.82	65.20	5.89 ± 3.84	34.38 ± 8.87	25.79	0.48 ± 0.12[illegible]

* Calculated as the mean ± SD of five technical replicates for each method, except for Method B that only had four for RNA assessments due to a technical error.

**Total yield was calculated based on the concentrations and the volume of eluates.

Table S7. Summary of quality assessments for DNA and RNA.

	DNA*		RNA**			
	**260/280 Mean ± SD**	**260/280 CV (%)**	**260/280 Mean ± SD**	**260/280 CV (%)**	**RIN Mean ± SD**	**RIN CV (%)**
Method A	2.03 ± 0.040	1.89	2.09 ± 0.010	0.40	7.34 ± 0.23	3.14
Method B	1.96 ± 0.050	2.31	2.14 ± 0.10	4.57	7.53 ± 0.46	6.17
Method C	1.94 ± 0.030	1.65	2.08 ± 0.020	1.00	7.02 ± 0.58	8.21

*DNA quality was assessed by NanoDrop (260/280 measurements) of the 5 technical replicates for each method.
**RNA quality was assessed by both NanoDrop and Bioanalyzer (RIN) of the 5 technical replicates for each method, except for Method B that only had four due to a technical error.

Table S8. The yield of protein (g/mg) and the total yield (mg) of brain tissue after analysis by the DC Protein Assay kit.

	**Concentration Mean* (mg/mL)**	**Concentration CV (%)**	**Total yield** (mg)**
Method A	18.48 ± 4.96	26.86	1.85 ± 0.50
Method B	18.52 ± 1.75	9.46	1.85 ± 0.18
Method C	23.52 ± 3.04	12.94	2.35 ± 0.30
Conventional	12.67 ± 0.84	6.59	12.67 ± 0.84

*Expressed as mean ± SD for five technical replicates for each extraction method.
**30 mg of ground tissue was used for multi-extraction methods (Method A, B, and C), whereas 50 mg was extracted using the conventional method.

Table S9. A list of abbreviations of oxylipin species and their internal standards.

Abbreviation	Compound name
d11-11(12)-EpETrE	d-11-11(12)-epoxyeicosatrienoic acid
d11-14,15-DiHETrE	d11-14,15-dihydroxyeicosatrienoic acid
d4-6-keto-PGF1a	d4-6-keto-prostaglandin F1 alpha
d4-9-HODE	d4-9-hydroxyoctadecadienoic acid
d4-LTB4	d4-Leukotriene B4
d4-PGE2	d4-Prostaglandin E2
d4-TXB2	d4-Tromboxane B2
d6-20-HETE	d6- 20-hydroxyeicosatetraenoic acid
d8-5-HETE	d8- 5-hydroxyeicosatetraenoic acid
12(13)-EpOME	12(13)-epoxyoctadecamonoenoic acid
13-HODE	13-hydroxyoctadecadienoic acid
13-oxo-ODE	13-oxo-octadecadienoic acid
9-HODE	9-hydroxyoctadecadienoic acid
9-oxo-ODE	9-oxo-octadecadienoic acid
9,10,13-TriHOME	9,10,13-trihydroxyoctadecamonoenoic acid
9,12,13-TriHOME	9,12,13-trihydroxyoctadecamonoenoic acid
9(10)-EpOME	9(10)-epoxyoctadecamonoenoic acid
20-HETE	20-hydroxyeicosatetraenoic acid
11-HETE	11-hydroxyeicosatetraenoic acid
11,12-DiHETrE	11,12-dihydroxyeicosatrienoic acid
11(12)-EpETrE	11(12)-epoxyeicosatrienoic acid
12-HETE	12-hydroxyeicosatetraenoic acid
12-oxo-ETE	12-oxo-eicosatetraenoic acid
14,15-DiHETrE	14,15-dihydroxyeicosatrienoic acid
14(15)-EpETE	14(15)-epoxyeicosatrienoic acid
14(15)-EpETrE	14(15)-epoxyeicosatrienoic acid
15-deoxy-PGJ2	15-deoxy-Prostaglandin J2
15-HETE	15-hydroxyeicosatetraenoic acid
15-oxo-ETE	15-oxo-eicosatetraenoic acid
20-COOH-LTB4	20-COOH- Leukotriene B4
20-OH-LTB4	20-OH-Leukotriene B4
5-HETE	5-hydroxyeicosatetraenoic acid
5-oxo-ETE	5-oxo-eicosatetraenoic acid
5,6-DiHETrE	5,6-dihydroxyeicosatrienoic acid
5(6)-EpETrE	5(6)-epoxyeicosatrienoic acid
6-keto-PGF1a	6-keto-prostaglandin F1 alpha
6-trans-LTB4	6-trans-leukotriene B4
8-HETE	8-hydroxyeicosatetraenoic acid
8,9-DiHETrE	8,9-dihydroxyeicosatrienoic acid
8(9)-EpETrE	8(9)-epoxyeicosatrienoic acid
9-HETE	9-hydroxyeicosatetraenoic acid

LTB4	Leukotriene B4
LTC4	Leukotriene C4
LTD4	Leukotriene D4
LTE4	Leukotriene E4
LXA4	Lipoxin A4
PGB2	Prostaglandin B2
PGD2	Prostaglandin D2
PGE2	Prostaglandin E2
PGF2a	Prostaglandin F2 alpha
PGJ2	Prostaglandin J2
TXB2	Tromboxane B2
12,13-DiHOME	12,13-dihydroxyoctadecamonoenoic acid
13-HOTrE	13- hydroxyoctadecatrienoic acid
9-HOTrE	9- hydroxyoctadecatrienoic acid
9,10-DiHOME	9,10-dihydroxyoctadecamonoenoic acid
PGD1	Prostaglandin D1
PGE1	Prostaglandin E1
10(11)-EpDPE	10(11)-epoxydocosapentaenoic acid
13(14)-EpDPE	13(14)-epoxydocosapentaenoic acid
16(17)-EpDPE	16(17)-epoxydocosapentaenoic acid
17-HDoHE	17- hydroxydocosahexaenoic acid
19(20)-EpDPE	19(20)-epoxydocosapentaenoic acid
7(8)-EpDPE	7(8)-epoxydocosapentaenoic acid
11(12)-EpETE	11(12)-epoxyeicosateteaenoic acid
12-HEPE	12-hydroxyeicosapentaenoic acid
14,15-DiHETE	14,15-dihydroxyeicosatetraenoic acid
15-HEPE	15-hydroxyeicosapentaenoic acid
17,18-DiHETE	17,18-dihydroxyeicosatetraenoic acid
17(18)-EpETE	17(18)-epoxyeicosateteaenoic acid
5-HEPE	5-hydroxyeicosapentaenoic acid
5,15-DiHETE	5,15-dihydroxyeicosatetraenoic acid
5,6-DiHETE	5,6-dihydroxyeicosatetraenoic acid
8-HEPE	8-hydroxyeicosapentaenoic acid
8,15-DiHETE	8,15-dihydroxyeicosatetraenoic acid
8(9)-EpETE	8(9)-epoxyeicosateteaenoic acid
PGD3	Prostaglandin D3
PGE3	Prostaglandin E3
Resolvin E1	Resolvin E1
LTB3	Leukotriene B3
15(S)-HETrE	15(S)-hydroxyeicosatrienoic acid

Supplementary Figures

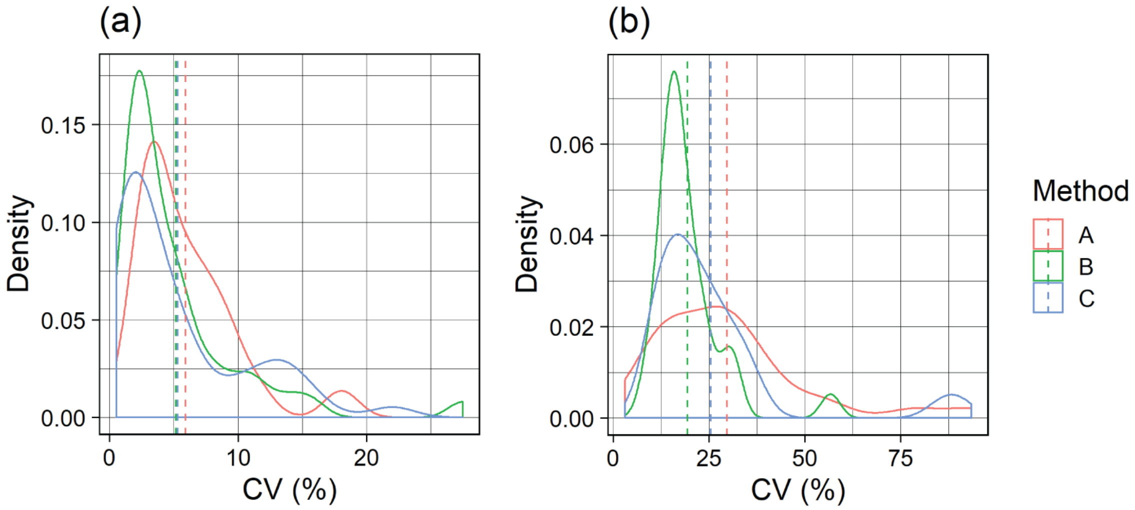

Figure S1. Density plots of the coefficient of variation (CV) calculated for (a) polar metabolites and (b) non-polar metabolites. CVs of the five technical replicates of each method were calculated for each metabolite, and used to generate the plot. Mean of all CVs within each group are denoted by the dotted lines. Method A (red), Method B (green), and Method C (blue).

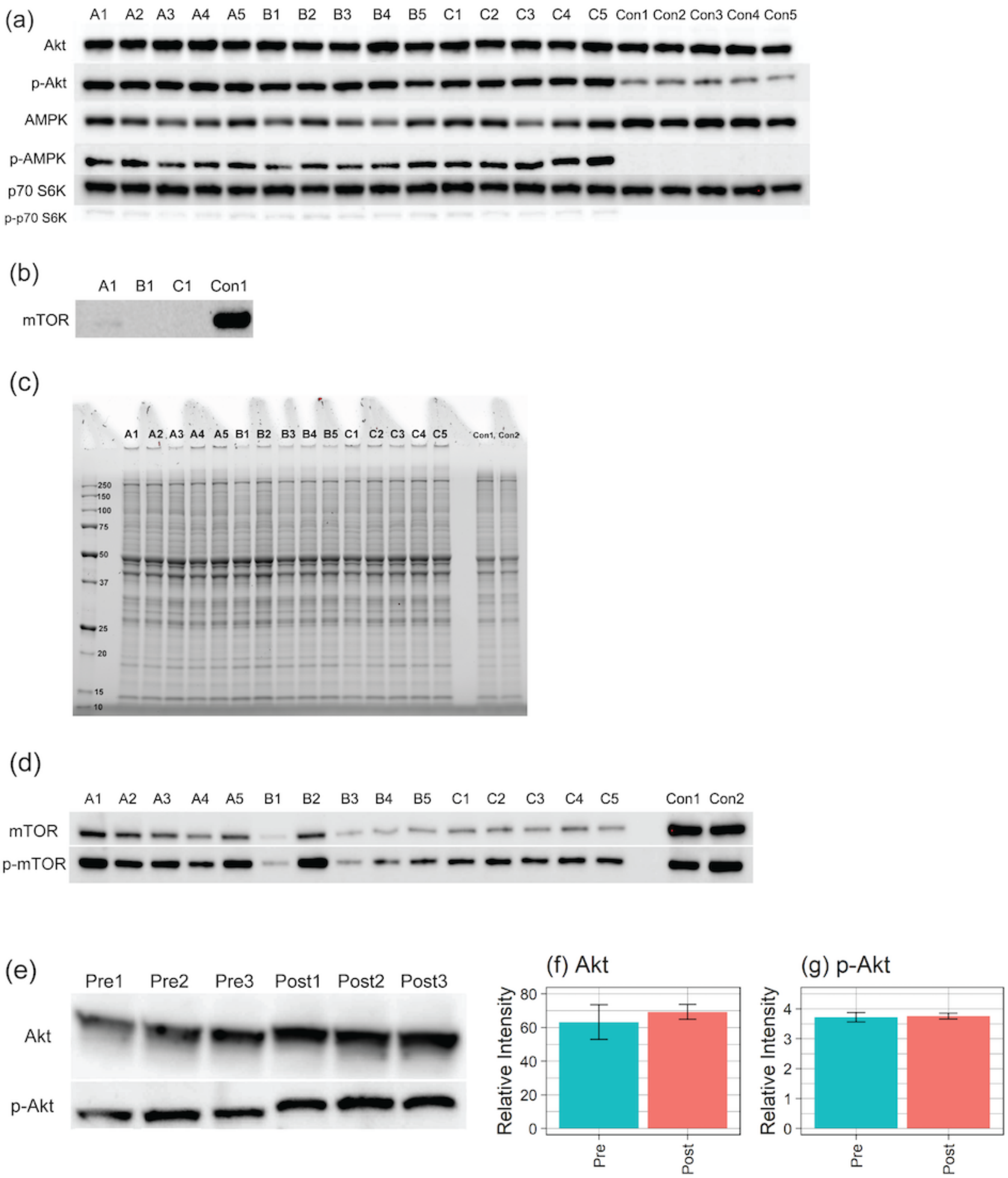

Figure S2. Comparison of protein recovery by Western blot before and after desalting. Chemiluminescent blots of (a) all proteins of interest except for mTOR before desalting and (b) mTOR before desalting treatment; (c) SDS-PAGE image after desalting; Chemiluminescent blots of (d) total and phospho-mTOR after desalting and (e) Akt and p-Akt pre- and post-desalting. Bar plots of the relative intensity of (f) Akt and (g) p-Akt before and after desalting. Protein samples extracted by the conventional method were not desalted.

Abbr: A1 - A5, replicates of Method A; B1 - B5, replicates of Method B; C1 - C5, replicates of Method C; Con1 - Con2, replicates of the conventional method; Pre1-Pre3, replicates of protein samples extracted by Method C and before applying desalting; Post1-Post3, replicates of protein extracted by Method C after desalting.
